# Rasping

**Published:** 1873-06

**Authors:** 


### RASPING.

A RASP is a rough file, applied to the hoof of a horse in shoeing, so as to make the edge of the hoof even with the edge of the shoe. The file is hard, the hoof is soft, hence it is rasped away with great rapidity.

Take a pin and move it along on the skin; repeat it one, two, or a dozen times, in the same identical track, the irritation becomes intense, unendurable, and convulsions follow.

There are a great many moral raspings and irritations in social and domestic life which wear the heart away, which wear off the better feelings of our nature, and if the soul is not made a wreck and a ruin at last, it is so scratched and marred as to be scarcely recognizable from what it was days agone. So happy and joyous it was then ; so broken and dispirited and sad is it now. When we sing

> " Blacksmith, very fine,
> Can you shoe this horse of mine ?"

it is easy to see who is the rasper ; but, in the moral world, it often happens that the man rasps himself ; all that is in him that is worth anything, is worn away by incessant fretting and worry and complaining and fault-finding. Nothing pleases him. He always wants the weather to be the reverse of what it is. Nobody does anything to suit him. He knows everything ; others nothing. He can tell you exactly what the President of the United States ought to do in any given case. He can inform A. T. Stewart how he could appropriate his spare millions to the highest possible good. He can tell his minister how to preach, so as to draw crowds to the building, and fill the communion table with new converts. If he had a wife he would "show her what's what ;" as for disciplining children, he could manage a whole houseful of them with perfect success ; as for servants, he would make them know their places and stay there ; and then, in all these diverse cases, when he sees a person acting differently from what he would have done, he gets so mad, " because they are fools, and have no sense," that he can scarcely contain himself ; and he dances around and snorts and fidgets and raves until he is almost beside himself.

And the ladies—bless their dear, thorny souls—how they do rasp their poor, weak "hubbies" into the grave sometimes. Why, I was witness to a lady's rasping her husband while he was dying. In his youth he learned to chew tobacco; she was the model of neatness and refinement, and the tobacco juice would sometimes find its way on the carpet, on his beard, on the corners of his mouth, and, O horror! on his shirt bosom. He chewed; she rasped. Finally, at the end of thirty years, he gave in, so far, at least, as to go out of doors when he wanted to chew, so as to chew in peace. But he was taken ill and lost all relish for tobacco, as is quite usual in serious sickness. The day before he died nature seemed to revive, and in the last desperate effort for life a feeble desire manifested itself for another chew, and he asked for a little tobacco. She became intensely alarmed, lest the old habit should return upon him, and began forthwith to rasp away with the old argument. During the process, he went off in the doze of debility, and the next day died.

Wives too often get into such a habit of rasping off the supposed hurtful excrescences of their husbands, that it becomes a second nature, and we have known them to keep at it after the man was dead.

But there is one great misfortune connected with "rasping;" it not only embitters the life of the rasper, but it wastes away and eats out the light and sunshine and joyousness of all who are within hearing. Father, mother, mistress, child, are you a domestic rasper? are you the moral pest of the household and the dinner table?

Dean Swift died demented; he had a great many irritations in life; but, besides this, he was of such a temperament that the contemplation of the weakness and folly of men often made him furiously angry. The inscription over his grave was, "Gone, where wrath against wrong-doing can no more tear the heart."

How beautiful the Scripture idea of

"THE OIL OF GLADNESS,"

a better remedy for the "sores" of humanity than a file.

